# Nonverbal dichotic test in patients with epilepsy

**DOI:** 10.1590/S1980-57642009DN30200007

**Published:** 2009

**Authors:** Karin Zazo Ortiz, Liliane Desgualdo Pereira, Alda Christina Lopes de Carvalho Borges, Luiz Celso Pereira Vilanova

**Affiliations:** 1 PhD in Neuroscience from UNIFESP, Professor at the Department of Speech Pathology and Audiology. Department of Speech Pathology and Audiology, UNIFESP, São Paulo, SP, Brazil.; 2,3 PhD in Human Communication Disorders, Professor at the Department of Speech Pathology and Audiology, UNIFESP, Department of Speech Pathology and Audiology, UNIFESP, São Paulo, SP, Brazil.; 4 PhD in Neurology. Professor at the Department of Neurology and Neurosurgery, UNIFESP, São Paulo, SP, Brazil.

**Keywords:** epilepsy, auditory perception, hearing disorders

## Abstract

Auditory processing during childhood may be altered if there is any predisposing factor during the course of development. Neurological disorders are among the risk factors for auditory processing disorders. Some studies have shown verbal auditory processing disorder in children with epilepsy.

**Objective:**

To verify the performance of children with epilepsy on a nonverbal dichotic test.

**Methods:**

Thirty-eight subjects, 23 female and 15 male, ranging from 7 to 16 years of age with neurological diagnosis of idiopathic epilepsy, without clinical or imaging evidence of cerebral lesion were evaluated. Patients were divided into two groups: 23 patients diagnosed with partial seizures and 15 patients with generalized seizures. Illiterate children, children with hearing thresholds exceeding the normal range and with brain lesions confirmed either clinically or by imaging tests were excluded from the study group.

**Results:**

Analysis of the performance of epileptic patients with partial and generalized seizures on the Nonverbal Dichotic Test revealed that the majority of patients with epilepsy showed impairments in the test, with no significant differences related to seizure type, generalized or partial. Although patients with partial and generalized seizures performed similarly, all the epileptic patients showed different performance to a normal population.

**Conclusions:**

This study revealed a high prevalence of impairments among epileptic patients in relation to nonverbal processing in a dichotic paradigm.

## Abstract (pt)

O processamento da informação auditiva na infância pode estar alterado se houver algum fator predisponente durante o desenvolvimento. As alterações neurológicas são um fator de risco para distúrbios no processamento auditivo. Alguns estudos já demonstraram alteração do processamento da informação auditiva verbal em crianças com epilepsia.

**Objetivo:**

Verificar a performance de pacientes com epilepsia no teste dicótico não-verbal.

**Métodos:**

38 crianças e adolescentes, na faixa etária compreendida entre 7 e 16 anos, com diagnóstico neurológico de epilepsia idiopática, 23 que apresentavam crise parcial e 15 com crise generalizada foram submetidas ao teste dicótico não-verbal.

**Resultados:**

Muitos pacientes com epilepsia apresentaram alterações no teste dicótico não-verbal. Não houve diferenças na presença e/ou tipo de alterações identificadas, segundo o tipo de crise, parcial ou generalizada.

**Conclusões:**

O estudo revelou alto índice de alterações no processamento auditivo de sons não-verbais, avaliado através de tarefa dicótica, em pacientes com epilepsia.

Auditory processing disorders involve deficits in the processing information in the auditory domain that are not due to higher order language, cognitive or other related factors.^1^ The evaluation of auditory processing can be carried out by means of several auditory tests. The dichotic listening paradigm was introduced by Broadbent^2^ and, following Kimura’s first studies,^3^ a large volume of research has been produced on this theme. Nonverbal dichotic tests were later introduced.^4^ Studies which involved musical chords and environmental nonverbal stimuli were also conducted.^5-7^

A great number of studies involving dichotic listening of nonverbal sounds aimed at investigating the lateralization effects of this kind of stimulus to cerebral hemispheres were carried out in brain damaged patients^8-11^ and normal subjects. Evidence emerged that neurological deficits^12-14^ are risk factors for impairments in auditory information processing. Therefore, children with epilepsy are at risk and could have auditory processing impairments, or at least, show differences in this processing compared with a normal population. Most of the research related to epilepsy dysfunction is based on the study of linguistic or cognitive abilities. However, some research was carried out based on auditory assessment.^14,15^ These investigations revealed differences in performance between epileptic and control groups, and sometimes differences were observed among epileptic patients, when factors such as seizure or epilepsy types are isolated. Other studies investigated auditory processing in children with temporal lobe epilepsy and observed that some tests were affected, especially verbal types.^13-15^ Bearing these issues in mind, the purpose of this study was to characterize the performance of patients with epilepsy on a nonverbal dichotic test, in order to check whether seizure type – partial or generalized – plays a role in the occurrence and type of disorder.

## Methods

This study was carried out at the Federal University of São Paulo, after being approved by the UNIFESP Research Ethics Committee. Thirty-eight children and adolescents, 23 female and 15 male, ranging from 7 to 16 years of age with neurological diagnosis of idiopathic epilepsy, without clinical or imaging evidence of cerebral lesion were evaluated. Patients were divided into two groups: 23 patients with partial seizures and 15 patients with generalized seizures.

Illiterate children, and children with hearing thresholds exceeding the normal range and those with brain lesions, confirmed either clinically or by imaging tests, were excluded from the study group. The selected subjects were submitted to a Nonverbal Dichotic Test. This test aims to verify a Selective Attention task using a binaural separation task, that is, the subject must pay attention to the sounds heard in one ear and ignore the sounds presented in the contralateral ear and point to the picture which represents the sound heard. For this test, we selected six nonverbal sounds, which were set in pairs in order to be presented simultaneously in one ear. These sounds represented a dog, a cat, a cock, a door shutting, a church bell and thunder. Onomatopoeic sounds were combined separately, since they have a different linguistic representation. Separating onomatopoeic sounds from the others, all possible permutations were made leading to 12 pairs of sounds. Sound pairs were presented at 50 dB HL. Subjects were first asked to point to one of the pictures representing one of the sounds heard and this condition was called Free Attention (FA). Subjects were next asked to point to the picture representing the sound heard in the right ear (AR) and finally, subjects were asked to point to the picture representing the sound heard in the left ear (AL).

The drawings representing the sounds are included in contextualized pictures.

In order to avoid calibration effects of the earphones, each condition of the test was carried out twice, reversing earphone placement on the second stimulation. Thus, each subject underwent each condition of 12 pairs of sounds twice, giving an overall stimulation of 24 dichotic sounds. Stimuli were presented using a MIDIMATE 622 audiometer coupled to a Sony CD Player.

Nonverbal Dichotic Test results from previously studied normal populations^6,7^ were used as standard for normal test performance. According to these parameters, in FA conditions there should not be predominance of one ear over the other, and the allowed difference between the recognition of both ears is one. Nevertheless, during forced attention conditions (AR and AL) the subject is expected to identify at least 23 sounds in the selected ear.

Hence, using the Nonverbal Dichotic Test we were able to establish the number of correct recognitions, which we called *correct responses*, and the number of wrong recognitions. In the latter condition, the subject may either fail to recognize the stimulus, which we called *error*, or point to the picture representing the contralateral stimulus during forced attention conditions which we called *reversal*.

Data analysis under FA, AR and AL conditions of the Nonverbal Dichotic Test was based on individual number of correct responses , errors and reversals in both ears.

The Mann-Whitney test and Fisher’s test were used. The significance level adopted was 5%.

## Results

Table 1 presents the mean values of errors (mean and standard deviation) under each condition of the Nonverbal Dichotic Test in a group of epileptic patients with partial or generalized seizures.

**Table 1:** Mean values of errors (mean and standard deviation) under each condition of the Nonverbal Dichotic Test, in a group of epileptic patients with partial or generalized seizures.

	Free attention (FA)			Attention to the right (AR)			Attention to the left (AL)	
	P	G		P	G		P	G
Mean	1.04	0.67		2.18	2.63		2.31	1.71
S.D	1.22	0.98		1.70	2.88		2.06	2.21
Mann-Whitney Test (P X G)	0.288			1.00			0.396	

P, Patients with partial seizure; G, Patients with generalized seizure.

Table 2 describes epileptic patients with partial or generalized seizures, according to the presence of right or left ear advantage in dichotic processing. This variable was elected for study because two previous studies have reported symmetric responses during FA,^6,7^ that is, half of the stimuli were identified in one ear and half in the contralateral ear, with an expected variation of only one stimulus. In the present study however, this pattern was not observed in 14 out of 23 patients with partial seizures, and in 10 out of 15 patients with generalized seizures. Based upon these results, we decided to investigate the presence of predominance of one ear over the other in both groups and where a difference was detected, whether one group differed significantly from the other.

**Table 2:** Predominance of right or left ear during FA in patients with partial or generalized seizures.

	REA			LEA			Total	
	N	%		N	%		N	%
P	6	42.9		8	57.1		14	100.0
G	5	50.0		5	50.0		10	100.0
Total	11	45.8		13	54.2		24	100.0

Fisher's Test; P=1.0000; REA, right ear advantage; LEA, left ear advantage; P, patients with partial seizure; G, patients with generalized seizure

Table 3 shows the performance of epileptic patients with partial or generalized seizure during AR and AL conditions of the Nonverbal Dichotic Test.

**Table 3:** Performance of epileptic patients with partial or generalized seizure during AR and AL conditions of Nonverbal Dichotic Test.

	AR			AL	
	N	A		N	A
P	5	18		10	13
G	7	8		8	7
Fisher's Test	0.15			0.74	

P, patients with partial seizure; G, patients with generalized seizure.

Table 4 illustrates mean values (mean and standard deviation) of reversals during AR and AL, respectively, on the Nonverbal Dichotic Test and shows statistical analysis of epileptic patients with partial and generalized seizures.

**Table 4:** Mean values (mean and standard deviation) of reversals during attention to the right ear and to the left on Nonverbal Dichotic Test.

	AR			AL	
	P	G		P	G
Mean	3.41	6.00		6.69	6.29
S.D	3.52	5.68		5.98	5.56
Mann-WhitneyTest (P X G)	0.282			0.936	

P, patients with partial seizure; G, patients with generalized seizure.

## Discussion

Study of the mean values of errors under each condition of the Nonverbal Dichotic Test in a group of patients with partial and generalized seizures revealed that both groups of epileptic patients showed a higher number of errors than that expected in a normal population.^6,7^ Moreover, the large standard deviation observed in this population seems to indicate that epileptic patients performed very differently from one another i.e. some had little and others great difficulty in performing tasks.

In this study, the errors were interpreted as a failure in stimulus identification during auditory-visual integration tasks. Many authors^16-18^ have described failures in visual and verbal memories in patients with epilepsy, while others^18^ claim that impairments in visual and auditory stimuli recognition are related to the poor initial decoding observed in epileptic patients. In fact, the Nonverbal Dichotic Test is extremely simple and easy to administer compared to other special tests used to assess auditory processing. This may suggest that the poor performance of epileptic patients may result from impairments in central visual and auditory processing or from problems during visual-auditory integration. Furthermore, during the test, pictures are part of a larger drawing. Subjects were required to analyze the picture thus constituting a more complex task than merely recognizing the picture. In normal subjects^7^ , context may have helped in the recognition process, while this was not the case in epileptic groups. Some authors have reported impairment in integration tasks in epileptic patients and described sites in the auditory pathway where interaction of sensory modalities take place, including visual modality.^12,18^ Table 2 describes epileptic patients with partial and generalized seizures, according to the presence of right or left ear advantage in dichotic processing. This variable was studied since symmetry of responses during FA was reported in earlier research, that is, half of the stimuli are identified in one ear and half in the contralateral ear, with an expected variation of only one stimulus. However, this pattern has not been observed in 14 out of 23 of our patients with partial seizures, and in 10 out of 15 our patients with generalized seizures. Based upon these results, it was necessary to investigate the presence of predominance of one ear over the other in both groups, and where a difference has been determined to check whether one group differed significantly from the other. There was no evidence of ear predominance during FA for both groups, with a similar number of identifications in the right and left ears, that is, when a lateralization effect was observed it occurred evenly to either the right or left ear. Some studies^6,18,19,20^ involving epileptic patients with partial seizures have shown that epileptic focus could facilitate the processing of auditory stimuli^19^ while lesion or attraction effects could also occur. In the lesion effect, stimulus processing takes place contralateral to the lesion^6^, or to the side of seizure occurrence in epileptic patients without lesions,^20^ whereas in the attraction effect, stimulus processing takes place in the non-compromised hemisphere where seizures take place.^21^ Since the seizure hemisphere variable was not studied in the group with partial epilepsy, either the lesion effect or attraction effect may have occurred, where any such effects would lead to the predominance of one hemisphere over the other. Furthermore, attentional basis may interfere with the asymmetry pattern.^21^ Other hypotheses include: morphological impairments, or an apparent “hyperfunctioning” of the hemisphere where seizures occur, as a result of changes in the interhemispheric balance due to the inhibition of the contralateral hemisphere or as a manifestation of changes in interhemispheric interactions.^21^ On the other hand, performance of patients with generalized seizure may be discussed based on the following hypothesis: inappropriate processing in subcortical regions or changes in interhemispheric balance.^22^ The issue of predominance of one hemisphere in this seizure type could be related to a greater compromise of subcortical structures^23^ which, as stated earlier, would provide balanced processing of nonverbal sounds. Regarding the changes in interhemispheric balance, as generalized seizures may lead to a greater number of structural impairments^18^ it is possible that the left hemisphere finished an action that had begun in the right hemisphere.^24-25^ It is noteworthy that as the procedure used in this study was the same as that employed in the normal population^6,7^ and the results in a normal population were different to those found in the present study, our results indicate impairments in both groups of epileptic patients.

Examination of the performance of epileptic patients with partial and generalized seizures in AR and AL (Table 3) showed a high incidence of impairments for both groups and demonstrated their difficulties in directed attention tasks.

Failure in stimuli identification or reversals were observed. The errors observed may be related to auditory and/or visual perceptual failures or failures in auditory-visual integration mechanism whereas reversals may be linked to auditory attention failures, predominance of one hemisphere in sound recognition and/or accomplishment of the task, or to corpus callosum impairments.

Table 4 illustrates mean values (mean and standard deviation) of reversals during AR and AL, respectively, on the Nonverbal Dichotic Test, and depicts statistical analysis of epileptic patients with partial or generalized seizures.

This variable was studied because it was necessary to check whether the subjects showed reversals in stimuli identification. Both groups had a similar number of reversals.

These findings suggest that patients with either partial or generalized seizures have difficulties directing their attention to one ear, and these difficulties are consistent with auditory attention failures or inability to maintain attention directed to one ear while ignoring the opposite. It was reported that the majority of central auditory tests require attention^26^ and that thalamus and thalamus-cortex circuit are critical to the selective attention process.^27^ Attentional mechanisms may interfere with auditory laterality.^28^ Brain stem efferent regulation plays an important role in dichotic perception of sounds^29^ and the left hemisphere is better prepared to select or focus the attention, whereas the right hemisphere is more adapted for selective attention or processing of different stimuli. Attention maintenance depends on normal functioning of subcortical structures.^30^ In patients with generalized seizures, primary impairment involves reticular formation in the brain stem. These researchers hold that thalamic structures function from the inhibition of thalamic nucleus which is controlled by reticular formation and the thalamic fronto-cortical system. These authors concluded that the main components of the activation system are reticular formation, medial thalamus and pre frontal cortex. Impairments in attention tasks have also been reported in patients with either partial or generalized seizure.^31^ These studies suggest that epileptic patients with partial or generalized seizure may have shown difficulties during selective attention conditions due to failures in attention maintenance. Attention is likely to be compromised in patients with generalized seizure due to brain stem impairments, and in patients with partial seizure due to subcortical compromise, and in both groups of patients due to impairments in subcortical pathways and central areas as well as corpus callosum dysfunction.

It was also noted that there was no predominance of one ear over the other for either group. This finding suggests that both groups could not sustain their attention for the selected ear, independently of seizure type. Occurrence of reversals during selective attention conditions suggest a deficit of auditory attention as opposed to a deficit in central decoding of sounds, since there was no ear predominance on stimuli identification.

Although the body of literature concerning epilepsy is vast, not many recent studies related to auditory processing of epileptic patients were found.

All data suggest that the occurrence of seizures, albeit partial or generalized, may cause structural and biochemical dysfunctions which compromise the proper functioning of the auditory pathway leading to sound processing impairments. However, there are many other factors that may influence the performance of patients with epilepsy in central auditory tests including: age at seizure onset, seizure frequency, cognitive impairment, emotional status, motivation and specific drug use. The extent to which these factors influence auditory processing should be the subject of further investigation in future studies.

In conclusion, studying the performance of epileptic patients with partial and generalized seizures on the Nonverbal Dichotic Test revealed that the majority of patients with epilepsy showed impairments on the test, without significant differences according to seizure type, albeit generalized or partial. Although the groups performed similarly, the performance of these epilepsy patients differed to that of a normal population. Furthermore, errors and predominance of one ear were observed during FA, without right or left ear advantage. During the Selective Attention tasks many reversals were found. These data suggest that the occurrence of seizures may represent a risk for auditory processing disorder impairments.
